# The “Making a Murderer” Case: A Brief Description on How EDTA Is Measured in Blood

**DOI:** 10.3389/fchem.2016.00041

**Published:** 2016-10-20

**Authors:** Steven R. Wilson, Luke Tolley

**Affiliations:** ^1^Department of Chemistry, University of OsloOslo, Norway; ^2^TranxendMapleton, UT, USA

**Keywords:** Making a Murderer, LC-MS, EDTA, mass spectrometry, liquid chromatography, Steven Avery

## Introduction

In 2007, Steven Avery was sentenced to prison for the murder of Teresa Halbach. What made this case stand out was that Avery had recently been released from prison, after wrongfully spending 18 years incarcerated on a rape charge. Advances in DNA analysis were used to prove Avery's innocence, resulting in his release. Avery quickly filed a lawsuit against Manitowoc County and former officials, whom Avery believed had framed him. In the midst of these procedures, Avery was again arrested for murdering Halbach. His nephew, Brendan Dassey, was also arrested and convicted as an accomplice.

The handling of the Halbach murder case was highly controversial. Steven Avery and his lawyers argued that they had he had once again been “set up” by his judicial adversaries. One central piece of evidence incriminating Avery was the presence of his blood at the crime scene. Avery's attorney's argued that the blood had been planted, arguing that the source was a test tube containing Avery's blood, which local police forces had access to.

But how determine if the crime scene blood was from a sample tube? The Avery tube contained ethylenediamine-tetraacetic acid (EDTA), which prevents blood coagulation and degradation. EDTA is not naturally present in human blood, and the defense argued that if EDTA was found in the crime scene blood, it would prove the blood was planted. The results of this analysis has generated vast discussion on social media and elsewhere, particularly following the documentary *Making a Murderer*^1^, which describes the life and trials of the Avery family. However, tools of forensics and chemical analysis are often “black boxes” to non-scientists, and perhaps early-stage science students as well. Even for the most curious and inquisitive, it can be difficult to learn more about these techniques, as they are mostly described in often-expensive scientific papers.

Therefore, we would like to here describe the central technique used for determining EDTA in blood spots in the Avery case, namely liquid chromatography-mass spectrometry (LC-MS). We here attempt to describe it in relatively simple terms. Please note that we will not discuss the judicial aspects of the case, but merely describe a central scientific technique used in a trial that has captivated and concerned millions of people.

## LC-MS: the measuring apparatus

LC-MS is a technique/apparatus that can be used for finding and measuring specific molecules in a sample. These molecules can be big (e.g., proteins) or small (e.g., amino acids), fatty or water-soluble. In other words, LC-MS is a versatile technique that can be used to search for a great variety of molecules.

Starting with the MS part: MS is an advanced instrument that is typically the size of a washing machine. MS measurements are used to calculate the mass of the molecules in a sample (Maher et al., 2015). A human being can have a mass of e.g., 75 kilos, but a single amino acid molecule has a mass of about 0.00000000000000000000000003 kilos. Hence, the MS is an extremely precise and accurate instrument that can be used to pinpoint a molecule's mass with great accuracy and precision. Therefore, if the MS detects a molecule corresponding to the mass of EDTA, there is already a fair chance that may be EDTA is present in the sample. Rather than kilos or pounds, the mass of a molecule is reported in daltons (Da); EDTA's exact mass is 292.09 Da.

There are many kinds of MS instruments (Maher et al., 2015). Some instruments determine the mass of a molecule by measuring the speed in which it flies in the MS, or measuring the frequency and radius in which it circles an electrode inside the MS.

However, other molecules present in a sample may have the same mass as EDTA^2^ http://www.chemspider.com/Molecular-Formula/C10H16N2O8, (Accessed September 2016). (just like many humans weigh 75 kilos; how could a balance tell them apart?). This presents a risk of a false positive, i.e., believing you are measuring one molecule, while in fact measuring another. Therefore, more steps can be taken to ensure a valid analysis. One is that the MS instrument can feature a *collision cell*, a chamber where molecules are bombarded with gas, breaking the molecule into smaller pieces. The masses of the pieces/fragments are then measured, and a computer visualizes the fragments of the shattered molecule. This visualization (called an “MS/MS spectrum”) can serve as a “fingerprint” of the molecule. If a mass fingerprint is for a particular molecule is observed, it serves as strong evidence that the molecule of interest is indeed present. An example of an EDTA MS/MS fragment is the mass 247.09, in which CO_2_ is chopped off the rest of the molecule. For more details on mass spectrometric features of EDTA (see e.g., Miller et al., 1997).

One problem with MS is that if you put a very complicated sample (e.g., blood) in the instrument, it gets “too crowded”; the MS might not be able to tell one molecule from the other, or have trouble detecting molecules (Annesley, 2003). In addition, one can be so unfortunate that two slightly different molecules may have virtually the same fingerprint (Wilson et al., 2010)!

**Figure 1:**
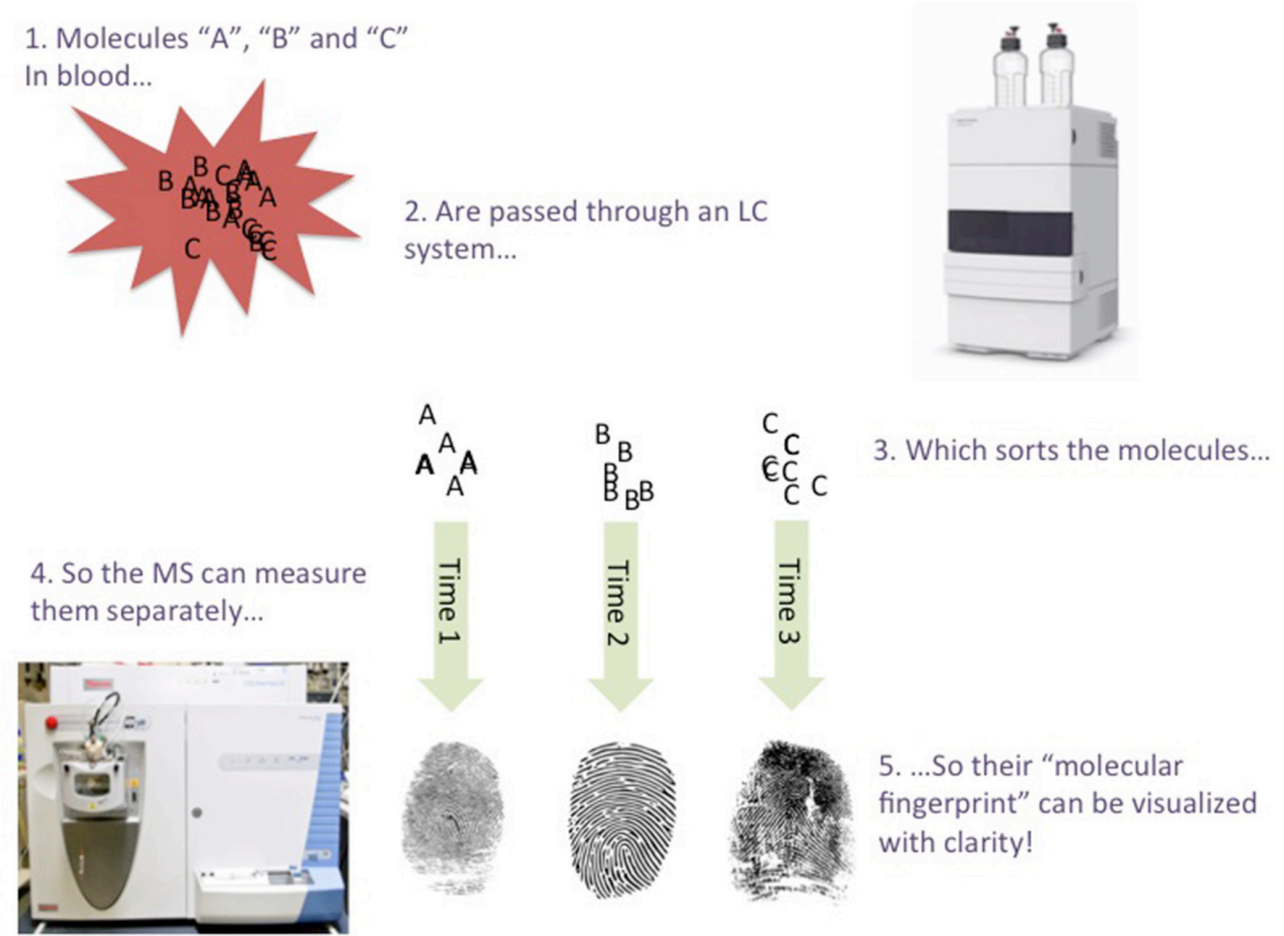
**Very simplified illustration of the LC-MS process**.

This is where the LC part comes in; LC (Snyder et al., 2011) is a technique that introduces different molecules from the sample to the MS at different time points, so the MS has fewer molecules to measure at a time. For example, the LC system may first permit very water-soluble molecules to enter the MS, followed by semi-soluble molecules, followed by poorly-soluble molecules, and so on. The LC forces the different molecules in a sample to “stand in a queue, to wait for their turn for entering the MS” (Figure 1). The time points in which a molecule enters the MS from the LC (called the *retention time*), are very precise, and can be used as additional grounds for identifying a molecule in a sample. An LC-MS analysis typically lasts for a few minutes or several tens of minutes, depending on e.g., the sample's complexity. If EDTA is present in a sample, its “mass fingerprint” should be detected, and at a specific retention time. When using LC-MS, an expert must manually analyze/confirm this “fingerprint”, although computer programs can assist (but not yet replace) this process. It is also important to compare a “fingerprint” found in a sample with a pure chemical. This is straightforward regarding EDTA, as it is commercially available at high purity.

Before the LC-MS analysis can begin, the proteins in the blood sample must be removed (proteins “stick to everything” and can ruin the LC-MS instrument parts). For measuring EDTA in blood, large proteins have typically been discarded using very fine filters (Johnsen et al., 2016).

## The avery samples

Regarding the Avery samples, the FBI was unable to detect EDTA using LC-MS^3^. It was concluded that EDTA was not in the samples analyzed.

When reading the recently released FBI reports on the analysis of Avery's blood^3^^,^^4^, it seems that the LC-MS part has all in all been suitably performed, with a number of steps taken to ensure that false positives and negatives would not occur, e.g., use of “mass fingerprint”, retention time, addressing that EDTA binds to metals, and use of an “internal standard” (Dolan, 2012) (adding and monitoring a “twin” molecule of EDTA, which is used to spot if anything has gone wrong with the analysis). The method does not appear to be “thrown together”, but is based on a previous method reported some years earlier (Miller et al., 1997). However, it would have been desirable if control samples absorbed to variety of absorbents (metal surfaces, wall paper, etc.) had been investigated, to demonstrate the validity and robustness of the total method.

Some limitations of the method are stated in the FBI reports. One weakness is that the system can “only” detect 0.013 mg of EDTA molecules per milliliter blood. In contrast, other compounds can be readily identified at 1000 times lower concentration or less. On the other hand, EDTA concentrations are is expected to be about 100 times higher than 0.013 milligrams of EDTA per milliliter blood (Miller et al., 1997).

The LC-MS spectra (the “fingerprint”) is not presented, making it difficult for viewers to acknowledge that there is a 100% certainty that EDTA was not present in the Avery blood stains. If these spectra were released for external inspection, it could relieve suspicions of contextual bias^5^. Another annoyance is that the report does not state the specific brand and specifications of the mass spectrometer used, which is a given to state in e.g., scientific papers.

## Author contributions

All authors listed, have made substantial, direct and intellectual contribution to the work, and approved it for publication.

### Conflict of interest statement

The authors declare that the research was conducted in the absence of any commercial or financial relationships that could be construed as a potential conflict of interest.

## Footnotes

^1^http://www.netflix.com/no-en/title/80000770, Accessed August 2016.

^2^http://www.chemspider.com/Molecular-Formula/C10H16N2O8, (Accessed September 2016).

^3^http://www.stevenaverycase.org/wp-content/uploads/2016/01/Trial-Exhibit-435-FBI-EDTA-Test.pdf, Accessed August 2016.

^4^http://www.stevenaverycase.org/wp-content/uploads/2016/01/Trial-Exhibit-434-Analysis-of-EDTA-in-Dried-Bloodstains.pdf, Accessed August 2016.

^5^http://www.post-gazette.com/opinion/Op-Ed/2016/03/13/The-Next-Page-Four-experts-explain-why-forensic-analysis-of-crime-scenes-is-not-as-reliable-as-you-might-think/stories/201603200003, Accessed August 2016.
